# Subacute *Staphylococcus epidermidis* Bacterial Endocarditis Complicated by Mitral-Aortic Intervalvular Fibrosa Pseudoaneurysm

**DOI:** 10.1155/2012/467210

**Published:** 2012-11-25

**Authors:** Diane Elegino-Steffens, Amy Stratton, Jone Geimer-Flanders

**Affiliations:** ^1^Department of Internal Medicine, Tripler Army Medical Center, 1 Jarrett White Road, Honolulu, HI 96819, USA; ^2^Department of Cardiology, Tripler Army Medical Center, 1 Jarrett White Road, Honolulu, HI 96819, USA

## Abstract

The patient is a 75-year-old man with a history significant for hypertension and congestive heart failure who underwent a bioprosthetic aortic valve replacement secondary to acute onset of aortic insufficiency. Cultures of the native valve were positive for *Staphylococcus epidermidis* sensitive to nafcillin and intravenous cefazolin was initiated. On postoperative day 24, he developed acute decompensated heart failure. A transesophageal echocardiogram demonstrated a structurally abnormal mitral valve with severe regurgitation, anterior and posterior leaflet vegetations, and scallop prolapse. There was also evidence of a mitral-aortic intervalvular fibrosa pseudoaneurysm (P-MAIF) with systolic expansion and flow within the aneurysm. Antibiotic treatment was changed from cefazolin to vancomycin for presumed development of methicillin-resistant *Staphylococcus*. He subsequently underwent a bioprosthetic mitral valve replacement and has restoration of health without sequella. This case highlights the development of a P-MAIF as a rare complication of both aortic or mitral valve replacement and infective endocarditis.

## 1. Introduction

The mitral-aortic intervalvular fibrosa (MAIF), also known as the mitral-aortic membrane, is a fibrous region of the heart that connects the anterior mitral leaflet to the posterior aortic root and communicates with the left ventricular outflow tract. The location of this segment and its involvement in the functional integrity between the mitral and aortic valve is clinically significant. As a consequence of its poor vascularization, this structure is the weakest segment of the aortic ring and is susceptible to injury during aortic or mitral valve replacement or destruction from infective bacterial endocarditis [1]. Damage can lead to development of an intervalvular fibrosa pseudoaneurysm (P-MAIF), which may cause rare and potentially life-threatening complications such as severe mitral regurgitation, rupture resulting in cardiac tamponade or hemoperitoneum, left ventricular fistula formation, coronary artery compression presenting as chest angina, pulmonary hypertension from pulmonary artery compression, and thrombosis causing a cerebrovascular accident or even death [2]. This is a case of an elderly male who initially underwent replacement of his aortic valve for severe aortic regurgitation that was subsequently found to have infective endocarditis prompting antibiotic treatment. His postoperative course was complicated by development of acute mitral regurgitation from a P-MAIF.

## 2. Case Presentation

The patient is a 75-year-old man with a medical history significant for hypertension and new onset congestive heart failure with acute severe aortic insufficiency who underwent aortic valve replacement with a bioprosthetic valve after presenting with progressive dyspnea and fatigue with minimal exertion. Preoperative transthoracic echocardiogram (TTE) demonstrated a left ventricular ejection fraction of 65–70%, severe aortic regurgitation with left ventricular dilation (a left ventricle end diastolic diameter of 6.8 cm and a left ventricle end systolic diameter of 4.6 cm) and was negative for valvular vegetations or mitral valve tethering. Cardiac catheterization was negative for coronary artery stenosis. His white blood cell count was 7.2 × 109/L, hemoglobin was 10.2 g/dL, hematocrit was 30.8%, platelet count was 151 × 109/L, blood urea nitrogen was 38 mg/dL, and creatinine was 1.2 mg/dL with a glomerular filtration rate of 55.9. Intra-operatively, he was noted to have an abnormal appearing aortic valve. On histologic examination of the native valve, it was found to have valvular necrosis with inflammation and gram positive cocci bacterial overgrowth that was consistent with infective endocarditis. Cultures of the aortic valve grew *Staphylococcus epidermidis* sensitive to nafcillin. He was subsequently treated with cefazolin (renally dosed for a GFR of less than 30%). His postoperative course was complicated by development of a third degree atrioventricular block for which he underwent pacemaker placement one week later. Placement of the pacemaker leads was performed using sterile technique and without evidence of a break in sterility.

**Figure 1:**
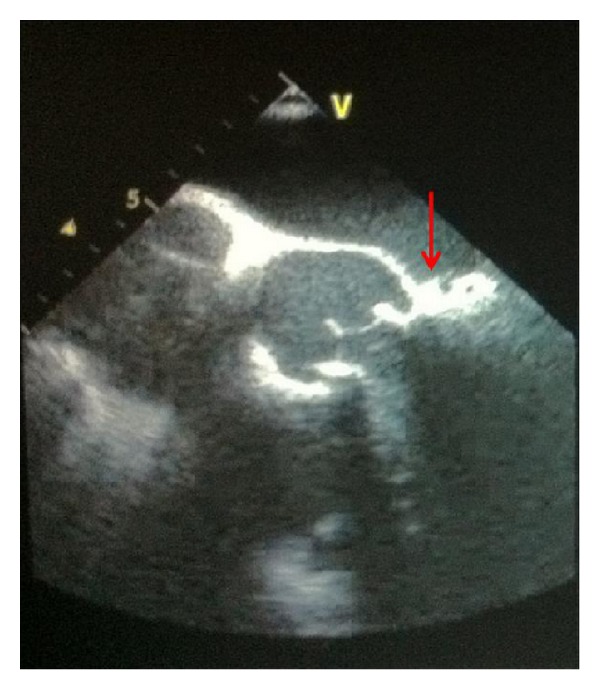
Transesophageal echocardiogram (TEE) showing the aortic valve, mitral valve, and mitral-aortic intervalvular fibrosa (red arrow) with adjacent pseudoaneurysm (P-MAIF).

**Figure 2:**
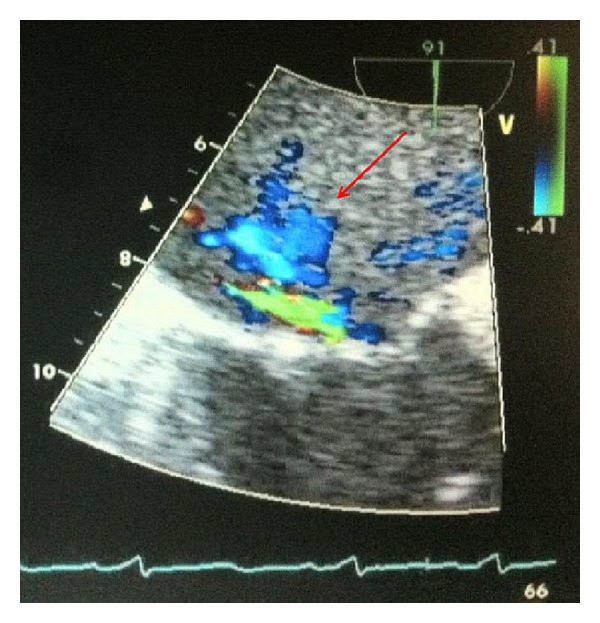
TEE showing scallop prolapse of the anterior mitral leaflet and severe mitral regurgitation (red arrow).

**Figure 3:**
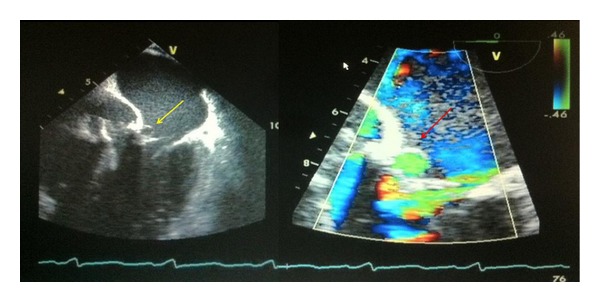
TEE depicting systolic ballooning of the aneurysmal segment of the P-MAIF (yellow arrow) and Doppler evidence of flow within the P-MAIF (red arrow).

**Figure 4:**
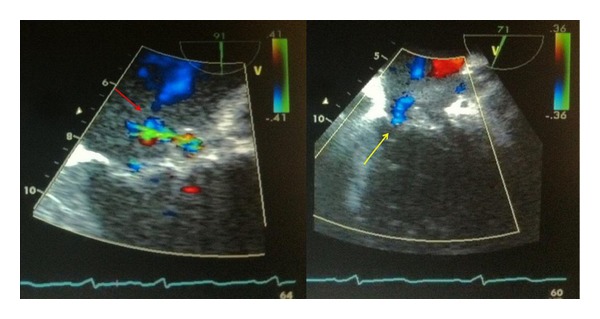
TEE of the mitral valve and P-MAIF demonstrating flow through the P-MAIF (red arrow) and shunt flow through the aneurysmal segment (yellow arrow) of the mitral-aortic intervalvular fibrosa (arrow head).

On postoperative day 24, he re-presented with worsening shortness of breath, atrial fibrillation/flutter, and evidence of acute decompensated heart failure. A transesophageal echocardiogram (TEE) was performed demonstrating a structurally abnormal mitral valve with acute severe regurgitation, anterior and posterior mitral valve leaflet vegetations, and a torn mitral valve cord with scallop prolapse (Figure 2). He was also found to have a P-MAIF with evidence of systolic expansion and flow within the aneurysm in addition to shunt flow through the aneurysmal segment of the MAIF (Figures 1, 3, and 4). There was no evidence of chordate rupture or oscillating mass on TEE. He was afebrile and had no symptoms consistent with infective endocarditis. However, blood cultures were positive methicillin-resistant *Staphylococcus epidermidis* and antibiotic treatment was changed to vancomycin for a total of 6 weeks.

He subsequently underwent mitral valve replacement with a bioprosthetic valve and currently has resolution of health without sequella. During the mitral valve replacement the bioprosthetic aortic valve was noted to be normal in appearance without evidence of infection. On histologic examination of the mitral valve, it was found to have gram positive cocci bacterial overgrowth that was consistent with infective endocarditis and concern for partially treated annular abscess. Cultures of the mitral valve demonstrated development of a methicillin-resistant bacteria matching the strain of *Staphylococcus epidermidis* found from the aortic valve and blood cultures. He subsequently completed a second 6-week course of intravenous vancomycin. He has since had restoration of health without further sequella.

## 3. Discussion

A P-MAIF is an anatomic poach between the medial wall of the left atrium and the aorta that communicates with the left ventricular outflow tract. Complications of a P-MAIF are rare but severe and include perforation, left ventricular fistula formation, cardiac tamponade, severe mitral regurgitation (as our patient developed), coronary artery or pulmonary artery compression, and death [2]. It is most frequently associated with aortic valve surgery and active or prior endocarditis [3]. Our patient had both a history of aortic valve endocarditis and recent aortic valve replacement.

Waldhausen et al. first described this entity in 1966 in a case involving a young boy who sustained a severe precordial trauma [4]. Since that time, a comprehensive review published by Sudhakar et al. in 2010 documented less than 100 patients with a P-MAIF after examining 5 case series and 62 case reports. In the absence of complications, patients with a P-MAIF are usually asymptomatic [3]. However, the most reported presentation was the evidence of an infection from active endocarditis [3]. Other clinical manifestations include shortness of breath, congestive heart failure, cerebral vascular accidents or other embolic events, and chest pain [3].

Diagnosis is made by visualization of systolic expansion and diastolic collapse of the P-MAIF, along with systolic turbulent flow seen on echocardiogram [5]. Diagnosis is more accurate with a transesophageal echocardiogram than with a transthoracic echocardiogram and the sensitivity may increase up to 90% from 43%, respectively [5]. Other testing modalities include coronary angiography to evaluate for coronary compression or atherosclerotic coronary disease and computed tomography or magnetic resonance imaging to evaluate the extent of a pseudoaneurysm and its effect on adjacent structures within the chest [3].

Management of a P-MAIF is an early surgical correction to prevent complications associated with a P-MAIF. The most employed surgical procedure is an aortic valve replacement. However, a variety of surgeries have been documented in the literature to include percutaneous pseudoaneurysm repair, mitral valve repair with coronary artery bypass, resection of the aneurysm, and heart transplantation [6]. In this case, despite aortic valve replacement and antibiotic therapy, the patient had further mitral valve destruction with resultant intervalvular fibrosa aneurysm and shunt flow through a tear in the aneurysmal segment with severe eccentric anterior mitral leaflet regurgitation. The patient required mitral valve replacement within two months from his initial aortic valve replacement. Furthermore, the early development of the third degree atrioventricular block in this patient's postoperative course may have been suggestive of an annular process with possible P-MAIF or annular abscess. It is important to note that as the patient underwent pacemaker placement there is always the possibility of bacteremia and endocarditis secondary to inducing infection during the procedure. However, in this patient this is unlikely due to the sterile fashion in which the leads were introduced and placed. Our case is unique in the fact that our patient presented without signs or symptoms of infective endocarditis and had bacterial involvement of both his aortic and mitral valves. This may also represent a case in which an annular abscess was partially treated and led to the development of a P-MAIF. This case has resulted in a change in the hospital policy at our institution to now include the requirement for peroperative transesophageal echocardiogram prior to all open heart surgeries, especially given the absence of evidence suggesting endocarditis in this patient.
